# Improved image reconstruction in coherent diffraction imaging using self-seeded XFEL pulses

**DOI:** 10.1107/S160057752600353X

**Published:** 2026-05-06

**Authors:** Jaeyong Shin, Junha Hwang, Sejin Kim, Sung Yun Lee, Eunyoung Park, Sangsoo Kim, Changyong Song, Daewoong Nam

**Affiliations:** a 4GSR Research Division, Pohang Accelerator Laboratory Pohang University of Science and Technology Pohang37673 Republic of Korea; b XFEL Beamline Department, Pohang Accelerator Laboratory Pohang University of Science and Technology Pohang37673 Republic of Korea; c Department of Physics Pohang University of Science and Technology Pohang37673 Republic of Korea; d Photon Science Center Pohang University of Science and Technology Pohang37673 Republic of Korea; e Center for Ultrafast Science on Quantum Matter, Max Planck POSTECH Korea Research Initiative Pohang37673 Republic of Korea; RIKEN SPring-8 Center, Japan

**Keywords:** coherent diffraction imaging, X-ray free-electron lasers, self-seeding, coherence, temporal coherence

## Abstract

In coherent diffraction imaging (CDI), the coherence properties of photons play critical roles in obtaining structural information from specimens without using lenses. While the impact of coherence has been widely studied in CDI, it has not been systematically investigated within the specific framework of X-ray free-electron laser (XFEL)-CDI. Here, we examined the relationship between the transverse and temporal coherence of XFEL pulses and the quality of image reconstruction using XFELs. Specifically, we investigated the properties of self-amplified spontaneous emission and self-seeding beams at an X-ray energy of 5 keV by collecting diffraction patterns from a single gold nanoparticle. Furthermore, the quality of the reconstructed images obtained using the two beam modes was compared. Our results demonstrate that the self-seeding beam offers more reliable image reconstruction that is attributable to the narrower bandwidth of the incident X-rays. This study highlights the advantages of utilizing a self-seeding beam in CDI experiments, particularly for enhancing the reliability and quality of image reconstruction.

## Abstract 2

This study demonstrates that the superior coherence of narrow-bandwidth self-seeded X-ray pulses significantly improves the quality and reliability of image reconstruction in coherent diffraction imaging compared with conventional broad-bandwidth SASE pulses. These findings highlight the critical role of spectral purity in achieving high-fidelity lensless imaging with X-ray free-electron lasers.

## 1. Introduction

Self-amplified spontaneous emission (SASE) is a key mechanism for generating extremely intense and coherent X-ray pulses using X-ray free-electron lasers (XFELs) (Bonifacio *et al.*, 1984 ▸; Ayvazyan *et al.*, 2002 ▸). The SASE beam is widely used to study the structural information of specimens in various fields, despite its naturally broad bandwidth (Seibert *et al.*, 2011 ▸; Chapman *et al.*, 2011 ▸; Ihm *et al.*, 2019 ▸; Barends *et al.*, 2022 ▸). To improve the energy resolution, a monochromator is employed, even at the cost of significantly reduced intensity, which is approximately 100 times weaker (Clark *et al.*, 2013 ▸; Shwartz *et al.*, 2014 ▸; Buzzi *et al.*, 2018 ▸; de Groot *et al.*, 2024 ▸; Fechner *et al.*, 2024 ▸; Bergmann *et al.*, 2021 ▸). As an alternative approach, self-seeding (SS), which offers a narrower bandwidth than the SASE beam does and a higher flux than monochromatic SASE does, has been introduced (Feldhaus *et al.*, 1997 ▸; Amann *et al.*, 2012 ▸; Inoue *et al.*, 2019 ▸; Rebernik Ribič *et al.*, 2019 ▸; Nam *et al.*, 2021 ▸). In addition, the transverse coherence of the SS beam was better than that of SASE (Pop *et al.*, 2022 ▸; Kim *et al.*, 2022 ▸).

Because of the coherent property and high flux of XFEL beams, coherent diffraction imaging (CDI) has garnered attention for revealing the structural information of non-crystalline specimens (Miao, 2025 ▸). A higher flux SASE beam with a relatively broad bandwidth is preferred for CDI experiments to achieve high-resolution imaging because the image resolution is related to the highest spatial frequency in the measured diffraction patterns. However, blurred features may appear at higher spatial frequencies in diffraction patterns because of the incoherent summation caused by the broad bandwidth of the SASE beam (Abbey *et al.*, 2011 ▸; Enders *et al.*, 2014 ▸; Huijts *et al.*, 2020 ▸; Malm *et al.*, 2020 ▸; Lin *et al.*, 2025 ▸). Previously, polychromatic CDI utilizing a specific algorithm was demonstrated to address broadband sources (Abbey *et al.*, 2011 ▸). However, applying this algorithm method to XFEL sources presents distinct challenges due to the inherent shot-to-shot fluctuation in the spectral properties of the SASE beam. That is, the quality of the diffraction patterns at high spatial frequencies is degraded owing to the poor temporal coherence of the SASE beam, which thereby hinders the phase retrieval process and makes it difficult to achieve good convergence. In contrast to other X-ray microscopy techniques, CDI is an indirect imaging method that does not rely on lenses. Instead, a phase-retrieval process is required to reconstruct real-space images from the collected diffraction patterns. When considering the entire process of obtaining real-space images in CDI, the phase retrieval transfer function (PRTF) is also suitable for evaluating image resolution because it directly reflects the effectiveness of phase retrieval (Chapman *et al.*, 2006 ▸).

In this study, we investigate the effect of the spectral bandwidth of incident X-rays on image reconstruction. The quality of image reconstruction was evaluated using the *R*-factor and PRTF. To obtain diffraction patterns with different bandwidths of incident X-rays, we used SASE and SS beams at the Pohang Accelerator Laboratory XFEL. The pulse energies and bandwidths of the SASE and SS beams at PAL-XFEL were approximately 2 mJ with a bandwidth of 20 eV [full width at half-maximum (FWHM)] and 0.9 mJ with a bandwidth of 0.41 eV (FWHM) at 9.7 keV, respectively (Nam *et al.*, 2021 ▸; Kim, Lee *et al.*, 2025 ▸).

## 2. Method

**Figure 1:**
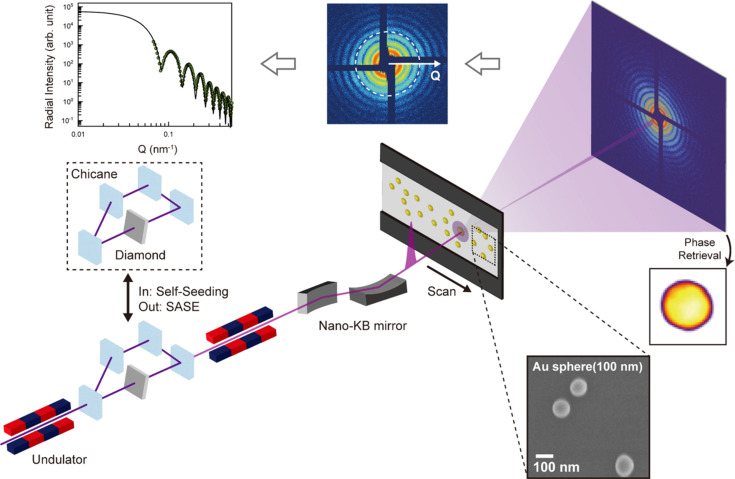
Conceptual schematic of coherent diffraction imaging experiments using nano-focused X-ray free-electron laser pulses. Femtosecond X-ray pulses were generated using the self-amplified spontaneous radiation process. Alternatively, spectrally narrower pulses were produced using a diamond crystal and a magnetic chicane (self-seeding scheme). The X-rays were focused to approximately 570 nm × 440 nm (FWHM) using Kirkpatrick–Baez mirrors. Gold nanoparticles with a diameter of 100 nm were dispersed on a silicon nitride membrane. Diffraction patterns from individual nanoparticles were recorded using a JUNGFRAU detector. Phase retrieval was applied to reconstruct 2D projected density maps of single nanoparticles. Additionally, the radial intensity distribution from each diffraction pattern was used to estimate the incident flux density and transverse coherence length when the specimen’s structural parameters were known.

**Figure 2:**
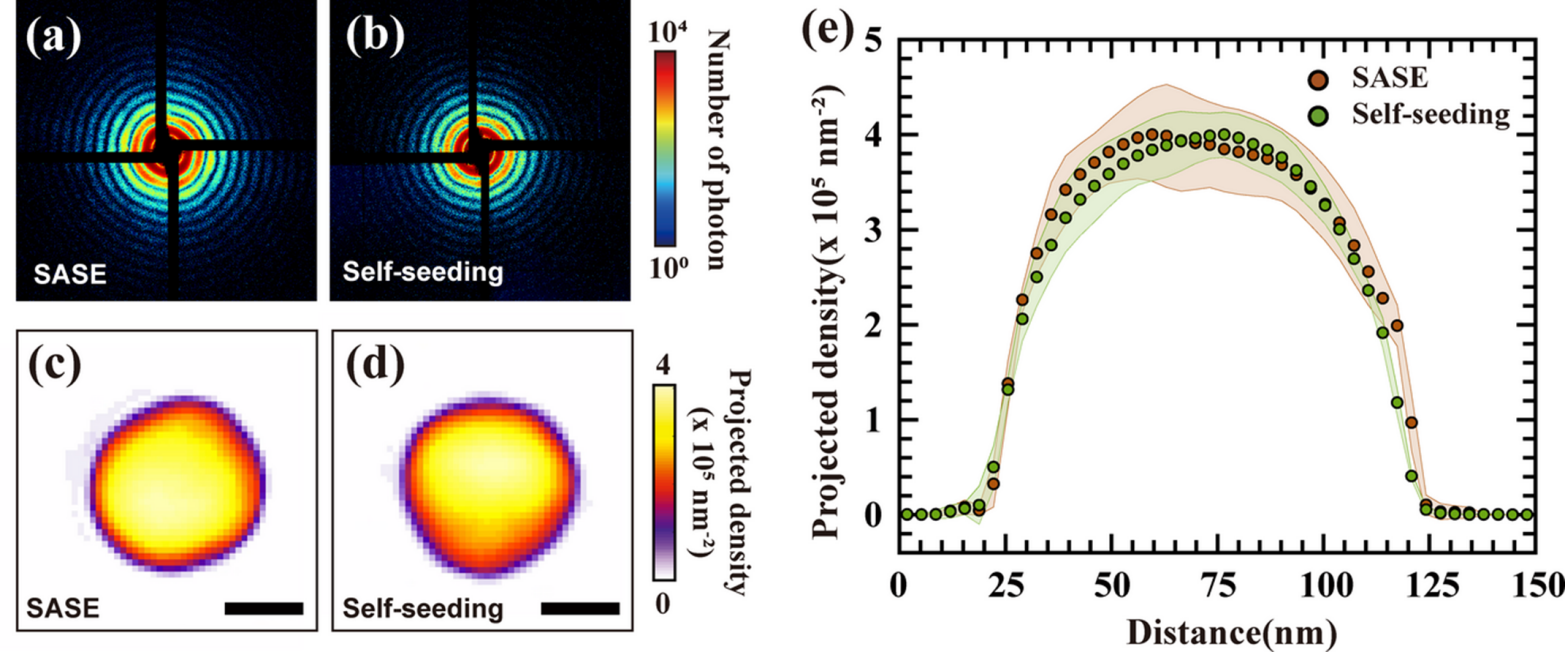
(*a*, *b*) Representative diffraction patterns of 100 nm-diameter gold nanoparticles obtained in a (*a*) SASE beam and (*b*) self-seeding beam. The colormap indicates the number of photons on a logarithmic scale. (*c*, *d*) Reconstructed projected electron density images corresponding to (*a*, *b*), respectively. Scale bars correspond to 50 nm in (*c*) and (*d*). (*e*) Line profiles of the horizontally projected density distributions from (*c*) and (*d*). Brown and green dots represent averaged profiles from the SASE and self-seeding beams, respectively. The shaded areas indicate the standard deviation of the 1D line profiles extracted 240 independent reconstructions.

We performed CDI experiments using a nanobeam X-ray experiment (NXE) instrument at the hard X-ray beamline PAL-XFEL (Kim, Hyun *et al.*, 2025 ▸). Fig. 1 ▸ shows a schematic of the CDI experiments. The accelerator components, including the undulators, were tuned to generate a SASE beam with a photon energy of 5 keV and a bandwidth of approximately 20 eV FWHM (see supporting information). When a magnetic chicane and a diamond monochromator were used, a self-seeded FEL with a sharp bandwidth of less than 1 eV was generated (see supporting information) (Nam *et al.*, 2021 ▸). The incident X-rays were focused to an area of approximately 570 nm × 440 nm using Kirkpatrick–Baez (K–B) mirrors. Because the specimens were evaporated by a focused single X-ray pulse, it was necessary to continuously supply fresh samples. A fixed target, such as an Si_3_N_4_ membrane, was used to supply fresh samples to each X-ray pulse via the raster scanning method. Gold nanospheres (nanoComposix, USA) with diameters of 100 nm were dispersed on the Si_3_N_4_ membrane. The nanoparticles were diluted in an ethanol solution to control their concentration. The Si_3_N_4_ membranes were plasma-cleaned to eliminate any impurities, and the nanoparticle solution was drop-cast onto the membranes, followed by spin-coating to ensure uniform particle distribution (Nam *et al.*, 2016 ▸). The 100 nm Au nanoparticles were specified by the manufacturer to have a size dispersion of ±3% (1σ). The diffraction patterns were recorded using an adJUstiNg Gain detector FoR the Aramis User (JUNGFRAU) station 4-megapixel (4M) detector. The detector was located approximately 800 mm from the interaction point and had a central hole of 3.3 mm × 3.3 mm to avoid radiation damage from the direct beam. Only the diffraction patterns of a single particle were selected, resulting in 207 and 227 diffraction patterns for the SASE and SSs, respectively. Figs. 2 ▸(*a*) and 2(*b*) show representative diffraction patterns acquired using the SASE and the SS beams, respectively. In both cases, the diffraction signals extend isotropically to high spatial frequencies, indicating high-resolution structural information.

Before retrieving the phase information of the diffraction patterns, all the diffraction patterns were binned by combining 3 × 3 pixels into a single pixel to improve the statistical signal-to-noise ratio. This process increases the photon counts per binned pixel and is particularly beneficial at high spatial frequencies, where the diffracted signal is weak. After binning, the linear oversampling ratio was approximately 8.8 in each dimension, which satisfies the oversampling criterion required for phase retrieval. Subsequently, all the diffraction patterns were cropped to 241 × 241 pixels. The generalized proximal smoothing (GPS) algorithm was processed using 240 independent reconstructions (Pham *et al.*, 2019 ▸). This process is repeated until the second generation, similar to a genetic algorithm, achieves better convergence (Chen *et al.*, 2007 ▸). The support was dynamically updated using the shrink-wrap algorithm during the zeroth generation and then fixed from the first generation onwards (Marchesini *et al.*, 2003 ▸).

## 3. Results

As described in the *Method* section[Sec sec2], representative reconstructed images corresponding to the diffraction patterns in Figs. 2 ▸(*a*) and 2(*b*) are displayed in Figs. 2 ▸(*c*) and 2(*d*), respectively. Each image displays the 2D projected electron density map of a spherical gold nanoparticle and was obtained by averaging 240 independent reconstructions. The black scale bars in Figs. 2 ▸(*c*) and 2(*d*) represent 50 nm. To evaluate reconstruction quality, we compared the 1D projected profiles of the reconstructed images. In Fig. 2 ▸(*e*), the brown and green dots represent the 1D projected profiles calculated from the reconstructed images obtained using the SASE and SS beams, respectively. The shaded areas indicate the standard deviation of the 1D line profiles extracted 240 independent reconstructions, reflecting the reconstruction-to-reconstruction variability. The line profile extracted from the reconstructed image using the SS beam shows reduced variability compared with the SASE beam, indicating improved reconstruction consistency. A quantitative comparison using the *R*-factor and PRTF is presented in the subsequent section.

### 3.1. Quality of image reconstruction of the SASE and SS operation modes

**Figure 3:**
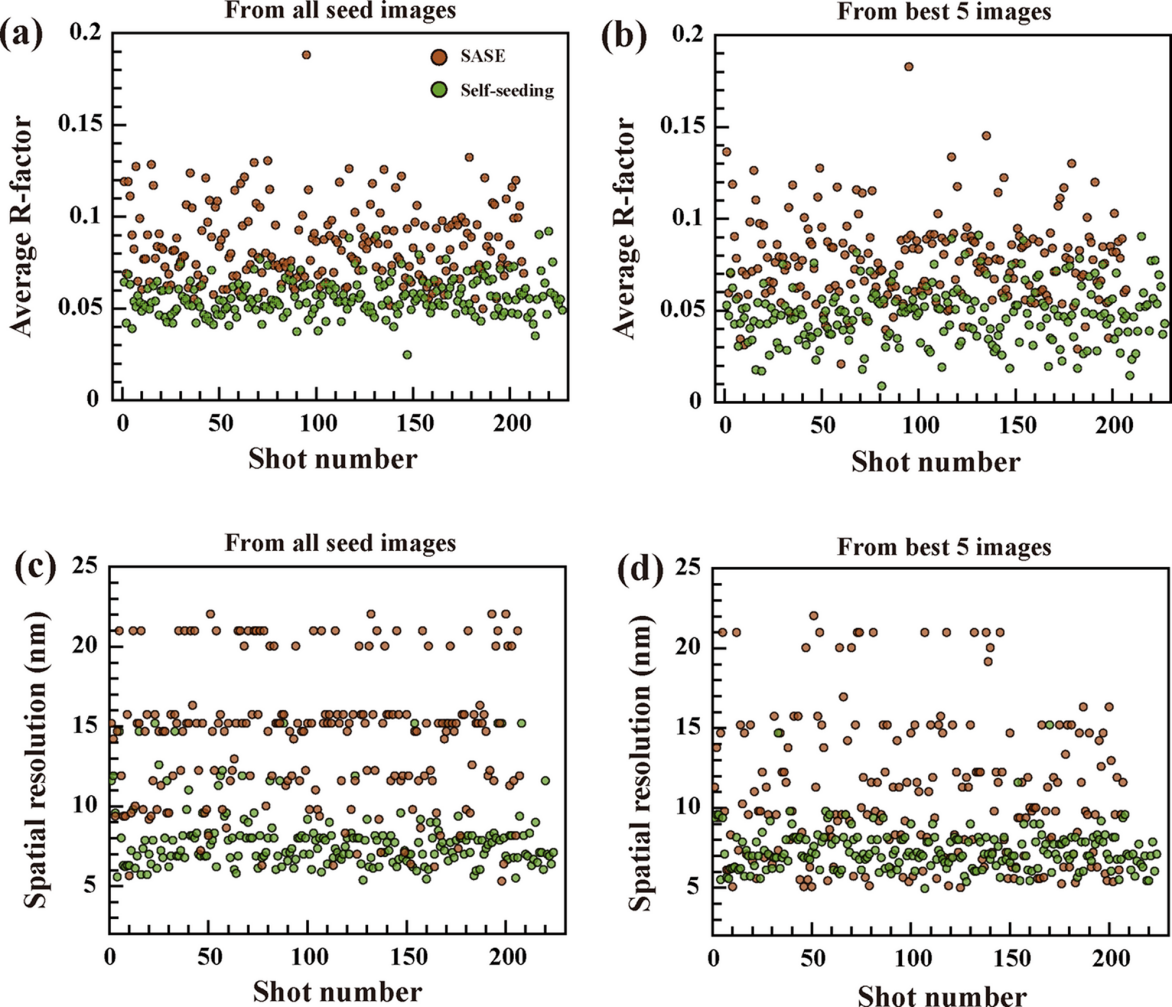
Comparison of the image reconstruction quality evaluated using *R*-factor and PRTF. (*a*, *b*) Average *R*-factor values obtained from all seed images (*a*) and from the five best images selected based on the lowest reconstruction error (*b*). Lower *R*-factor values indicate higher consistency between reconstructed images. Brown and green points represent results from the SASE and SS beams, respectively. (*c*, *d*) Spatial resolution estimated from the PRTF, calculated from all images (*c*) and from the five best images (*d*) after alignment and averaging. The resolution was determined based on the 0.5 PRTF criterion. In both evaluations, the SS beam consistently demonstrated superior reconstruction reliability and higher spatial resolution compared with the SASE beam.

We compared the quality of the reconstructed images acquired in the SASE and SS beams using the *R*-factor and the PRTF (see supporting information). Both metrics are widely used in CDI experiments to evaluate the reliability of reconstructed images. The *R*-factor and PRTF were obtained from each diffraction pattern using two sampling methods. We analyzed all 240 reconstructed images obtained from each diffraction pattern using a phase-retrieval process. In this case, the *R*-factor was calculated by comparing all 240 reconstructed images, and the average *R*-factor was computed, as presented in Fig. 3 ▸(*a*). For the PRTF calculation, all images were first aligned and then averaged. Subsequently, the PRTF was calculated, as shown in Fig. 3 ▸(*c*). In the second method, we selected the five best images from 240 reconstructed images. The best images were selected based on the lowest error, which was calculated as the difference between the measured and reconstructed diffraction patterns. In CDI, a representative image is typically obtained by averaging the best five or ten images of all the reconstructions. The *R*-factor was calculated using only the five best images, and the average *R*-factor is shown in Fig. 3 ▸(*b*). Similarly, for PRTF, a representative image was obtained by averaging the five best images after alignment. The PRTF for each diffraction pattern was calculated as shown in Fig. 3 ▸(*d*). This dual approach, which considers all reconstructed images and selects the best five, provides a comprehensive evaluation of image reconstruction reliability in CDI experiments.

First, the *R*-factors of the reconstructed images obtained in each operation mode were examined. The *R*-factor was calculated by comparing each reconstructed image. A lower *R*-factor score indicates a closer resemblance between the images. Figs. 3 ▸(*a*) and 3(*b*) show the results of the *R*-factor calculated using all 240 reconstructed images and the five best images, respectively. Each point indicates the average value of the calculated *R*-factors, with brown and green points representing the SASE and SS beams, respectively. In both results, the average *R*-factor values obtained using the SS beam were less than 0.1. When considering all the images from each reconstruction, 28.2% of the SS results scored below 0.05. For the best five images, 56.4% of the SS results scored below 0.05. In contrast, for the SASE beam, 79.7% of the 207 cases considered all images, and 86.5% of the five best images scored less than 0.1. For scores below 0.05, only one case was observed for all images, and 7.2% of the five best images achieved this score. Additionally, the distribution of the averaged *R*-factor in the SS beam exhibited a lower average value and smaller standard deviation than those observed in the SASE beam. These results indicate that the consistency of the numerically reconstructed images was more remarkable when self-seeded FEL pulses were used, further highlighting the benefits of the SS beam in the CDI experiments at XFELs.

We also compared the PRTF results obtained by comparing the radial summation of the measured diffraction pattern with that of the calculated diffraction pattern. Generally, a PRTF score of 0.5 is widely used as the criterion for determining the image resolution in CDI experiments. Figs. 3 ▸(*c*) and 3(*d*) show the PRTF scores obtained from both operation modes for the whole seed and five best images. Similar to the results of the *R*-factor calculation, the reconstructed images obtained in the SS beam exhibited better image resolution than those obtained in the SASE beam, based on the 0.5 criterion. Because diffraction from a spherical specimen exhibits periodic intensity minima, the diffracted intensity and the corresponding PRTF values can drop sharply at these specific spatial frequencies. Consequently, the image resolution defined by the PRTF is highly sensitive to such minima. To mitigate this sensitivity and avoid potential bias in the resolution estimate, we additionally performed a smooth PRTF analysis (see Fig. S3 in the supporting information) (Ekeberg *et al.*, 2024 ▸). Based on both evaluation methods, the reconstructed images from the SS beam demonstrated more reliable results and better image resolution with less deviation. Specifically, the SS reconstructions achieved resolution of 7.98 ± 2.07 nm (PRTF) and 7.60 ± 1.50 nm (smooth PRTF), outperforming the SASE reconstruction, which yielded 14.32 ± 4.15 nm (PRTF) and 12.20 ± 4.10 nm (smooth PRTF). These results indicate that the diffraction patterns calculated from the SS reconstruction closely resemble the measured diffraction patterns at high spatial frequencies. Occasionally, the SASE beam showed better image resolution when the PRTF was calculated using the five best images [Fig. 3 ▸(*d*)]. However, the overall behavior of the PRTF score in the SS beam exhibited greater stability, highlighting the reliability of the image reconstruction achieved with self-seeded FEL pulses. Thus, the reconstructed images obtained in the SS beam were more reliable than those obtained in the SASE beam.

### 3.2. Relationship between transverse coherence and quality of image reconstruction

The improvement in the image reconstruction achieved by the SS beam is related to the differences between the properties of the SASE and SS beams. To investigate this, we first examined the effects of the transverse coherence of each XFEL pulse on the *R*-factor and PRTF.

**Figure 4:**
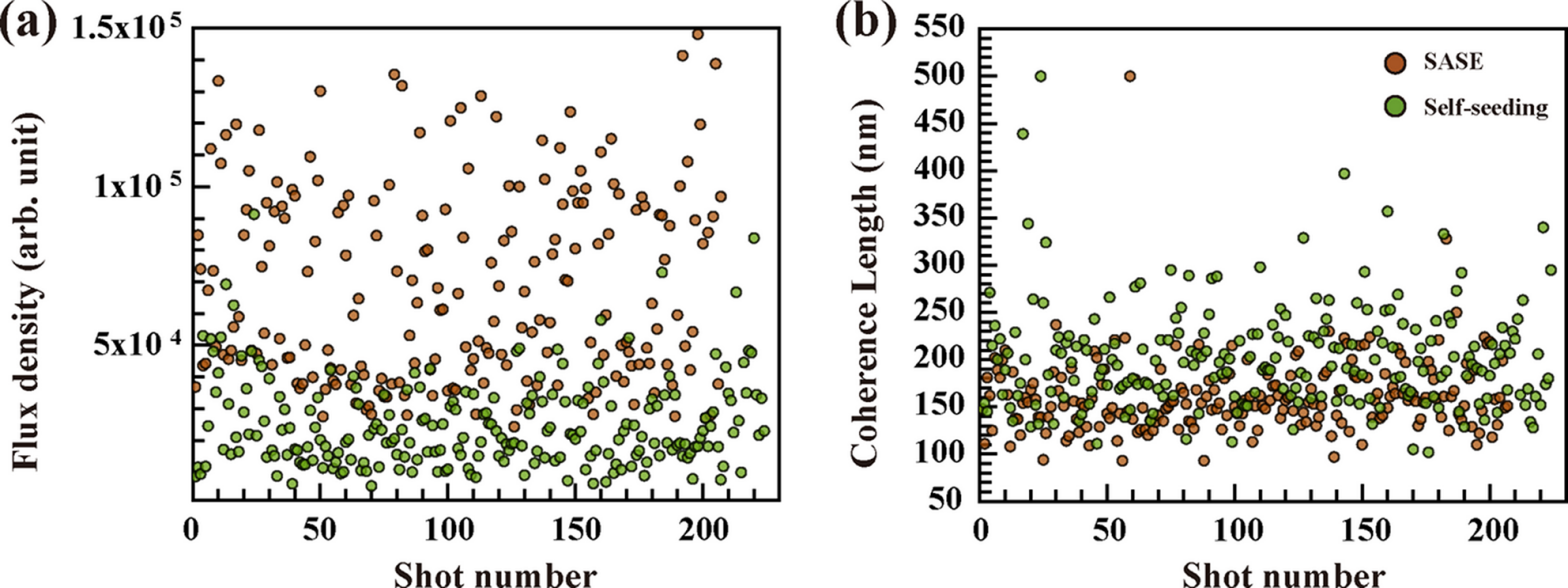
Illuminated flux density and transverse coherence length estimated from diffraction patterns of single gold nanoparticles. These values were deduced by comparing the radial intensity distribution curves with a fitted curve based on the ideal spherical model. (*a*) Estimated flux density illuminating the sample. (*b*) Transverse coherence length. Brown and green dots represent data from the SASE and self-seeding beams, respectively. The SASE beam exhibits approximately twice the flux density compared with the self-seeding beam, whereas the self-seeding beam shows slightly higher transverse coherence length than the SASE beam.

When the size, shape, and elemental composition of a specimen are known, the properties of the X-ray pulse can be inferred from the measured diffraction patterns. The illuminated flux density on the sample and transverse coherence length can be estimated (Lee *et al.*, 2020 ▸). Fig. 4 ▸ summarizes the estimated photon flux density and transverse coherence length for both beams. As expected, the photon flux density incident on the sample in the SASE beam was higher than that in the SS beam [Fig. 4 ▸(*a*)]. It is approximately twice that of the SS beam. In contrast, the transverse coherence of the SS beam was greater [Fig. 4 ▸(*b*)]. These results quantitatively demonstrate the transverse coherence length estimated using the fitting process. The overall distribution of the SS beam (green points) is greater than that of the SASE beam (brown points), with an average coherence length of approximately 202 nm for the SS beam and 162 nm for the SASE beam. The SS beam, characterized by its longer transverse coherence length, is consistent with observations from previous studies (Pop *et al.*, 2022 ▸; Kim *et al.*, 2022 ▸).

**Figure 5:**
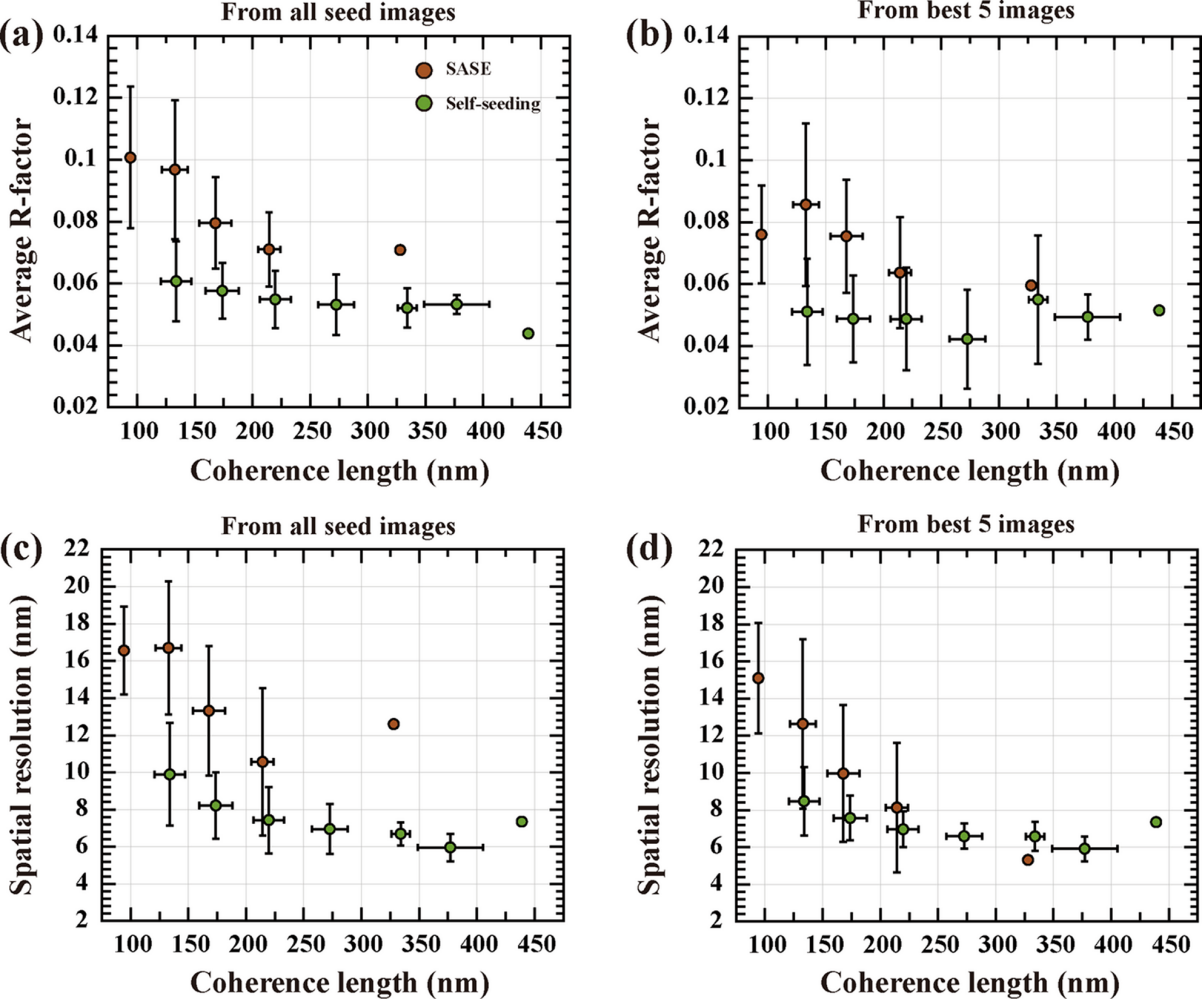
Relationship between the transverse coherence length and image reconstruction quality evaluated by *R*-factor and PRTF. The data were grouped into 50 nm bins along the *x*-axis to highlight trends and reduce statistical fluctuations. Brown and green points represent results from the SASE and SS beams, respectively. (*a*, *b*) Average *R*-factor as a function of transverse coherence length calculated using all seed images (*a*) and the five best images (*b*). (*c*, *d*) Spatial resolution determined by the 0.5 PRTF criterion for all images (*c*) and the five best images (*d*). In both evaluations, greater transverse coherence lengths correlated with improved reconstruction quality. The SS beam consistently yields lower average values and smaller standard deviations than the SASE beam, indicating higher reliability and better convergence in image reconstruction. The horizontal error bars indicate the standard deviation of the transverse coherence length within each 50 nm bin.

We investigated the relationship between the transverse coherence length and image reconstruction quality, as estimated by the *R*-factor and image resolution obtained by the PRTF using the 0.5 criterion (Fig. 5 ▸). To clearly illustrate this correlation, we grouped the data into 50 nm bins along the *x*-axis instead of displaying scattered data points. This representation enhances the trend analysis and minimizes statistical fluctuations to provide a more interpretable visualization of the relationship between the transverse coherence length and image reconstruction quality. In Fig. 5 ▸, the brown points indicate the SASE beam, whereas the green points represent the SS beam. Figs. 5 ▸(*a*) and 5(*b*) show the averaged *R*-factor results obtained using all reconstructed images and the five best images. In both the SASE and SS beams, the average *R*-factor decreased as the transverse coherence length increased, indicating that higher transverse coherence contributed to more consistent and reliable image reconstruction. Similarly, for the image resolution estimated by the PRTF with a score of 0.5, a better image resolution was achieved with increasing transverse coherence length, further confirming the positive impact of transverse coherence on image reconstruction quality [Figs. 5 ▸(*c*) and 5(*d*)]. Notably, the average *R*-factor and image resolution acquired using the PRTF in the SS beam exhibited a lower standard deviation and average compared with the SASE beam, as shown in Fig. 5 ▸. These results suggest that the SS beam provides more reliable and good convergence image-reconstruction. Hence, we conducted additional investigations to further understand the reason for this lower standard deviation in the SS beam.

### 3.3. Relationship between temporal coherence and quality of image reconstruction

We examined the effect of the spectral bandwidth of incident X-ray pulses on the quality of image reconstruction through numerical simulations. Diffraction patterns of an ideal sphere with a diameter of 100 nm were generated with varying bandwidths: a perfectly monochromatic case and three polychromatic cases with FWHM bandwidths of 10, 20 and 30 eV. All the diffraction patterns were defined on the same reciprocal-space (*q*-space) scale as the measured experimental data. In addition, we assumed that the incident X-ray flux was identical across all cases to ensure consistency and eliminate potential variations in the flux as a contributing factor to the observed differences in the results.

For each polychromatic case, diffraction patterns were generated by incoherently summing multiple monochromatic patterns weighted by a Gaussian spectral distribution centered at 5 keV. For the 10 eV case, 21 monochromatic patterns were generated at 0.8 eV intervals within the ±2σ range. A similar procedure was followed for the 20 and 30 eV cases (see supporting information). All simulated patterns included a missing central area of 48 × 48 pixels corresponding to the beam stop region of the detector. The simulated patterns incorporated both a photon counting noise and detector readout noise. The detector readout noise was applied at the level of 2 ADC counts (σ), while the photon counting noise followed a Poisson distribution according to the photon number at each pixel.

**Figure 6:**
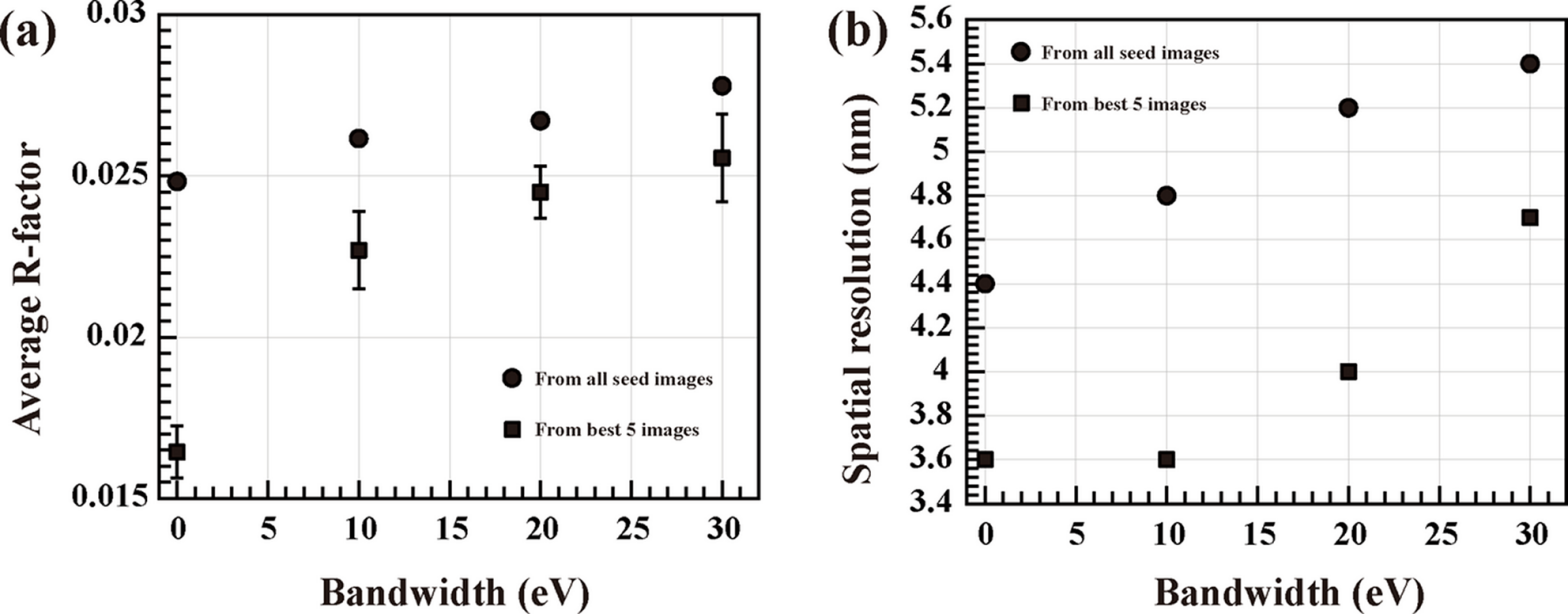
Numerical simulation results investigating the effect of temporal coherence as a function of energy bandwidth. (*a*) Average *R*-factor as a function of bandwidth for all seed images (circles) and the best five images (squares). The vertical error bars for the best five images represent the standard deviation of the pairwise *R*-factor values (ten combinations) computed among these five images. (*b*) Spatial resolution determined using the PRTF with a score of 0.5. The resolution limit of 3.6 nm is imposed by the detector size in this simulation. Both metrics show degradation in reconstruction quality with increasing bandwidth, highlighting the importance of narrow bandwidth for high-fidelity imaging. Error bars indicate the standard deviation.

Prior to phase retrieval, the diffraction patterns were preprocessed following the same protocol used for the experimental data, including 3 × 3 pixel binning. The phase retrieval was conducted using identical procedures. From the reconstructions, we computed the *R*-factor and the PRTF to quantify reconstruction quality. Fig. 6 ▸(*a*) shows the *R*-factor results, where each point represents the average *R*-factor across all initial phase retrieval seeds. For the best five images, the vertical error bars represent the standard deviation of the pairwise *R*-factor values calculated from the ten possible combinations among these selected images. The average *R*-factor was lowest for the monochromatic case and increased progressively with bandwidth. This trend was also evident when considering only the five best-reconstructed images per case, indicating that broader spectral bandwidths degrade reconstruction accuracy. For the PRTF analysis, two averaged images were computed for each bandwidth: one from all reconstructed images and another from the five best ones. The corresponding PRTFs, shown in Fig. 6 ▸(*b*), indicate that higher image reliability at high spatial frequencies was maintained when the incident bandwidth was narrower. As the bandwidth increased, the resolution estimated by the PRTF deteriorated in both averaging cases. This degradation is attributed to the blurring of high-frequency diffraction signals due to the incoherent summation of wavefronts with varying photon energies.

## 4. Discussion and conclusion

We investigated the properties of SASE and SS beams by analyzing the diffraction patterns of single particles. Through this investigation, we determined the illuminated flux density on the sample and the transverse coherence length of each beam. The analysis revealed that, while the flux density of the SASE beam is approximately double that of the SS beam, the transverse and temporal coherence properties of the SS beam are superior. This enhancement in the coherence properties of the SS beam is consistent with previous experimental and simulation studies (Osaka *et al.*, 2019 ▸; Pop *et al.*, 2022 ▸; Kim *et al.*, 2022 ▸). Specifically, the monochromator used to filter photon energies in the undulator line effectively functions as a spatial mode filter, contributing to this improvement. Furthermore, the reconstructed images obtained using the two beams were compared to evaluate the impact of these beam properties on reconstruction quality. We observed that a higher transverse coherence length enhanced the consistency and reliability of the image reconstruction as evaluated by the *R*-factor and the PRTF. This trend was consistently observed across both beam modes. Consequently, the reconstructed images from the SS beam yielded more reliable results and an overall higher spatial resolution with reduced deviation. These improvements are attributable to the synergistic effect of the narrower bandwidth and the enhanced transverse coherence facilitated by the spatial filtering of the SS beam. These results were further supported by simulation studies. This study highlights the advantages of using an SS beam to conduct CDI experiments at XFELs. Specifically, the improved beam quality in terms of overall coherence led to superior image reconstruction fidelity with more stable quantitative metrics. Given that cavity-based XFELs (CBXFELs), the next generation of X-ray sources, are designed to produce highly coherent X-rays with narrow bandwidths, our findings suggest that CDI experiments at these advanced facilities could achieve even better spatial resolution. Furthermore, such capabilities will likely attract significant attention for high-resolution structural studies of specimens.

## Supplementary Material

Sections S1 and S3, and Figs. S1 to S3. DOI: 10.1107/S160057752600353X/yi5190sup1.pdf
