# Supplementary material for: Improved image reconstruction in coherent diffraction imaging using self-seeded XFEL pulses
Source: J Synchrotron Radiat. 2026 May 6;33(Pt 4):904–12. doi: 10.1107/S160057752600353X (PMC13344604; doi:10.1107/S160057752600353X)
Supplement: Supplementary file 1 [file s-33-00904-sup1.pdf]

JOURNAL OF SYNCHROTRON RADIATION

Volume 33 (2026)

Supporting information for article:

Improved image reconstruction in coherent diffraction imaging using self-seeded XFEL pulses

Daewoong Nam, Jaeyong Shin, Junha Hwang, Sejin Kim, Sung Yun Lee, Eunyoung Park, Sangsoo Kim, Changyong Song and Daewoong Nam

S1. R-factor and Phase Retrieval Transfer Function

The R-factor and phase retrieval transfer function (PRTF) are fundamental metrics used to evaluate the quality of reconstructed images. The average R-factor is defined as:

$$\text{Averaged R} - \text{factor} = \frac{\sum_{i>j} \left|\frac{\rho_i - \rho_j}{\rho_i + \rho_j}\right|}{(N^2 - N)/_2}$$

where ρ_i and ρ_j indicate the reconstructed images from different seeds and N represents the total number of independent reconstructions. This quantifies the consistency of the reconstructed images. A smaller R-factor indicates better similarity among all images, suggesting that the phase retrieval algorithm produces reliable and good convergence image-reconstruction. In contrast, the PRTF was calculated by comparing the measured diffraction pattern with the calculated pattern, defined as:

$$\text{PRTF}(q) = \frac{|FT\{< \rho >\}(q)|}{\sqrt{I_M(q)}},$$

where q is a point of the reciprocal scape, $\text{FT}\{< \rho >\}(q)$ is the Fourier transform of the averaged reconstructed images, and $I_M\ (q)$ is the measured diffraction pattern. The PRTF measures the reliability of the reconstructed phases in reciprocal space and is used to estimate the image resolution. A threshold, typically 0.5, was applied to the PRTF to determine the image resolution in the CDI.

S2. Numerical Simulation

Four diffraction patterns of an ideal sphere with a diameter of 100 nm were generated with varying bandwidths: one perfectly monochromatic and three polychromatic cases with bandwidths of 10, 20, and 30 eV (FWHM). The central X-ray energy was set to 5 keV in all the cases. All the diffraction patterns were defined on the same q-space scale as the measured patterns. In addition, we assumed that the incident X-ray flux was identical across all cases to ensure consistency and eliminate potential variations in the flux as a contributing factor to the observed differences in the results. Diffraction patterns for each energy bandwidth were generated as an incoherent summation of multiple monochromatic diffraction patterns. The intensities of the monochromatic diffraction patterns were assumed to follow a Gaussian distribution:

$$\text{I}(q_x, q_y) = I_0 \sum_{E=-2\sigma}^{2\sigma} P(q_x, q_y, E) e^{(E/_{2\sigma^2})}.$$

Here, I_0 represents the maximum intensity of the diffraction pattern obtained from the experiment, and P denotes the monochromatic diffraction pattern for each energy. For example, in the case of a diffraction pattern with a bandwidth of FWHM 10 eV, a total of 21 monochromatic diffraction patterns were incoherently summed, generated at 0.8 eV intervals within the ±2σ range of the energy spectrum. For FWHM values of 20 and 30 eV, 43 and 63 monochromatic diffraction patterns were incoherently summed, respectively.

The simulation process began with the generation of an ideal polychromatic diffraction pattern for a given energy bandwidth. This ideal pattern was then scaled so that the photon count at the central peak was I_0. Subsequently, to mimic the experimental beam stop, a central area of 48 × 48 pixels was removed. Finally, a two-step noise was applied. First, photon shot noise was introduced by replacing the ideal photon count at each pixel with a random integer drawn from a Poisson distribution ($\lambda = I_{ideal}$). Second, detector readout noise was added by sampling from a Gaussian distribution with a standard deviation of σ=2 ADC counts to complete the final pattern.

In our simulations, the spectral bandwidth effect was modeled as Gaussian broadening of the diffraction patterns without explicitly including the stochastic spiky structure of individual SASE spectra. While this simplification neglects shot-to-shot spectral fluctuations, we note that in our experimental analysis more than 400 single-shot diffraction patterns were included. Thus, the statistical averaging effectively mitigates these fluctuations, making the Gaussian approximation a reasonable representation for evaluating the overall impact of bandwidth on CDI reconstructions.

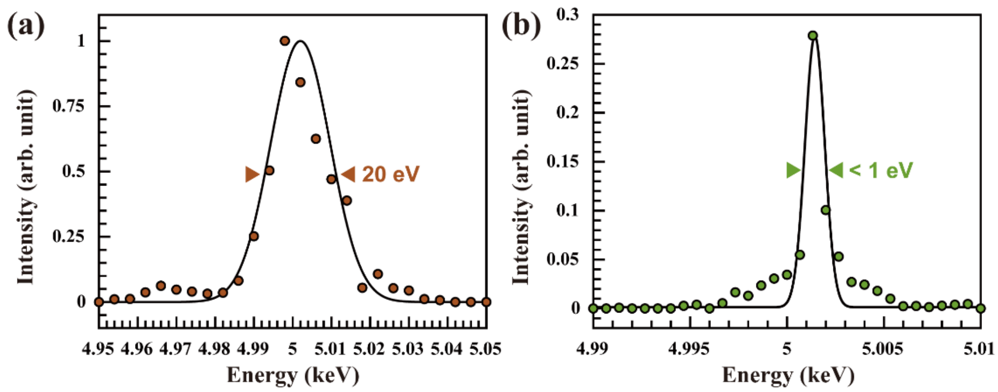

Figure S1 Typical spectral bandwidth of the SASE (a) and self-seeding (b) beams at PAL-XFEL. Both spectra are centered around 5 keV, with bandwidths of approximately 20 eV for the SASE beam and less than 1 eV for the self-seeding beam. The spectra were measured using a silicon double-crystal monochromator (DCM) located in the optical hutch of the hard X-ray beamline. For the self-seeding beam, the resolution of the Si DCM is insufficient to fully resolve the sub-eV bandwidth. The bandwidth of the self-seeding beam was estimated using the bent crystal reported in (Kim, Lee *et al.*, 2025).

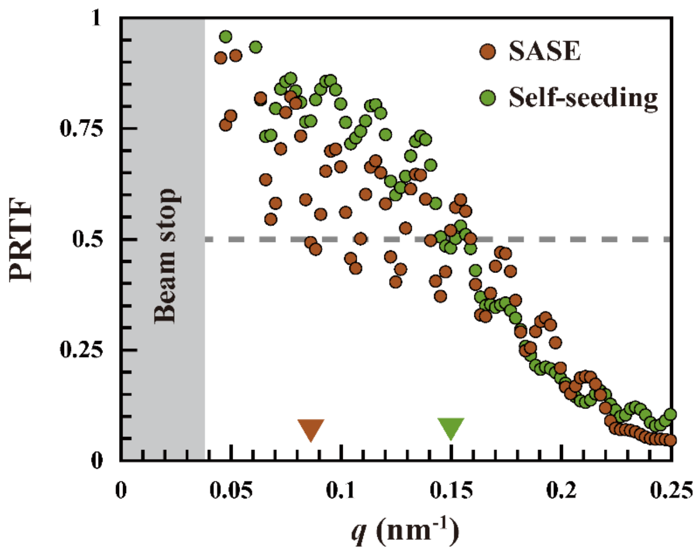

Figure S2 To complement the representative reconstructions shown in Fig. 2, we provide the corresponding PRTF curves. The PRTF 0.5 criterion gives $q \approx 0.087$ nm^{-1} (11.5 nm) for SASE beam and $q \approx 0.15$ nm^{-1} (6.7 nm) for self-seeding beam, confirming that self-seeding beam reconstructions preserve higher phase reliability at high spatial frequencies.

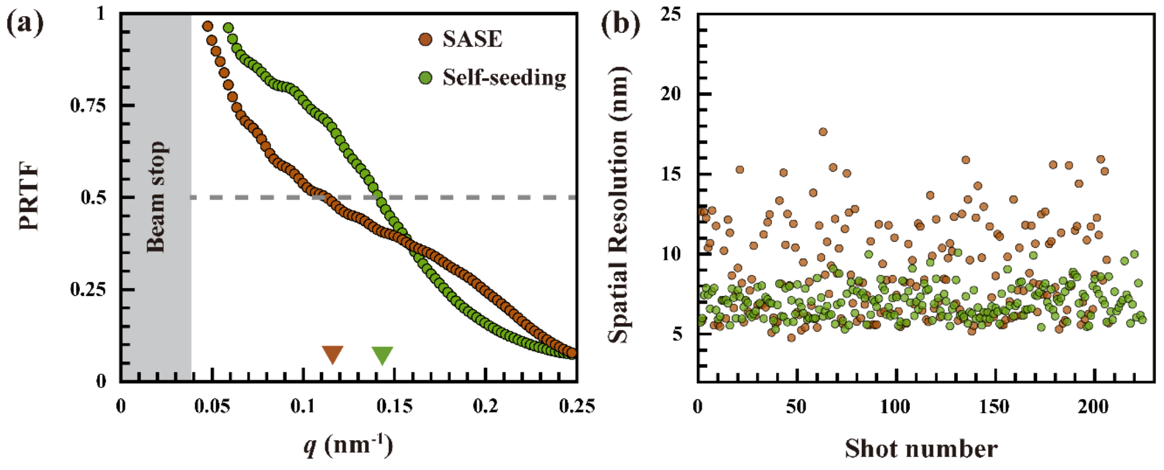

Figure S3 (a) Representative smooth PRTF curves corresponding to the diffraction patterns shown in Fig. 2, obtained by applying a Gaussian filter to the radial PRTF profiles. (b) Spatial resolution estimated from the smooth PRTF using the 0.5 criterion for all diffraction patterns. Brown and green points represent results from the SASE and self-seeding (SS) beams, respectively. The SS beam yields a resolution of 7.6 ± 1.5 nm, compared to 12.2 ± 4.1 nm for the SASE beam.
